# Two *Synechococcus* genes, Two Different Effects on Cyanophage Infection

**DOI:** 10.3390/v9060136

**Published:** 2017-06-02

**Authors:** Ayalla Fedida, Debbie Lindell

**Affiliations:** Faculty of Biology, Technion—Israel Institute of Technology, Haifa 32000, Israel; aya.shlos@gmail.com

**Keywords:** cyanophage, marine *Synechococcus*, host-virus interactions, host defenses, stress-response genes, gene inactivation, burst-size, PIN-PhoH

## Abstract

*Synechococcus* is an abundant marine cyanobacterium that significantly contributes to primary production. Lytic phages are thought to have a major impact on cyanobacterial population dynamics and evolution. Previously, an investigation of the transcriptional response of three *Synechococcus* strains to infection by the T4-like cyanomyovirus, Syn9, revealed that while the transcript levels of the vast majority of host genes declined soon after infection, those for some genes increased or remained stable. In order to assess the role of two such host-response genes during infection, we inactivated them in *Synechococcus* sp. strain WH8102. One gene, SYNW1659, encodes a domain of unknown function (DUF3387) that is associated with restriction enzymes. The second gene, SYNW1946, encodes a PIN-PhoH protein, of which the PIN domain is common in bacterial toxin-antitoxin systems. Neither of the inactivation mutations impacted host growth or the length of the Syn9 lytic cycle. However, the DUF3387 mutant supported significantly lower phage DNA replication and yield of phage progeny than the wild-type, suggesting that the product of this host gene aids phage production. The PIN-PhoH mutant, on the other hand, allowed for significantly higher Syn9 genomic DNA replication and progeny production, suggesting that this host gene plays a role in restraining the infection process. Our findings indicate that host-response genes play a functional role during infection and suggest that some function in an attempt at defense against the phage, while others are exploited by the phage for improved infection.

## 1. Introduction

Marine unicellular cyanobacteria belonging to the genus *Synechococcus* are highly abundant in the oceans, where they play a major role in primary production and carbon fixation [1,2]. They are constantly exposed to infection by phages which impact their population dynamics by killing a fraction of the population on a daily basis (estimated to be between 0.005% and 30% daily) [3,4,5]. Cyanophages are also thought to greatly impact the diversity and evolution of their cyanobacterial hosts [6,7,8,9,10,11,12].

One abundant cyanophage group in the oceans comprises the T4-like cyanophages, tailed double-stranded DNA phages that resemble the T4 coliphage archetype, both in virion morphology and core gene content [13,14,15]. Syn9 is a representative of this group and has a relatively broad host range [4]. It infects multiple *Synechococcus* strains that belong to different phylogenetic clades, occupy different ecological niches, and differ in the gene content of their flexible genome [1,16]. 

Recently, we found that Syn9 underwent a near identical infection and transcriptional program in multiple *Synechococcus* hosts (*Synechococcus* sp. strains WH8102, ,WH8109, and WH7803), despite the above-mentioned differences [16]. In response to Syn9 infection, the transcript levels of the vast majority of host genes (>90%) in each of the three hosts declined significantly [16]. However, transcript levels of a small group of host genes increased or remained unchanged during the phage latent period, and are considered host-response genes [16]. While these genes belong to the same general function groups in the different hosts (cell envelope, DNA repair, carbon fixation, respiration, and nutrient utilization), the actual genes are highly host-specific, making up part of the flexible genome, with many located in hypervariable genomic islands in their respective hosts [16]. This phenomenon is not unique to infection by Syn9. Indeed, a similar response was found during the infection of *Prochlorococcus* MED4 by the T7-like cyanophage, P-SSP7 [8]. Furthermore, other bacteria also display the upregulation of a limited number of host-response genes after phage infection [17,18,19,20]. 

Little is known about the functional role of these host-response genes during the interaction with the infecting phage. Some of them may serve as host stress-response genes, while others may constitute a host attempt at defense against phage infection. Alternatively, they may be induced by the phage for its own needs. Here, we began testing these hypotheses by investigating the impact of the independent inactivation of two host-response genes in *Synechococcus* sp. strain WH8102 (referred to from here as *Synechococcus* WH8102) on the Syn9 infection process. We chose two genes that may be involved in mounting a host defense, seen by the presence of potential host defense-related domains according to homology-based annotation. Both of the genes are the first in two-gene operons and thus, the two genes in each operon may have related activities that function together. 

The first two-gene operon is SYNW1659 and SYNW1658. The SYNW1659 gene consists of a domain of unknown function, DUF3387, that is often associated with restriction enzymes [16], a well-known mechanism of defense against phages [21], as well as with helicases, which is itself a common domain in restriction enzymes. This gene will be referred to as a DUF3387 gene from here on. The SYNW1658 gene consists of a different domain of unknown function (DUF1651) that is also found in other host-response genes in *Synechococcus* sp. strains WH8102 and WH8109 [16], in addition to *Prochlorococcus* sp. strain MED4 [8]. The transcript levels of these genes increased in response to infection by Syn9 in *Synechococcus* WH8102 [16]. 

The second two-gene operon may form a toxin-antitoxin module [22,23], which is also a known anti-phage defense mechanism [21,24,25]. The first gene in the operon, SYNW1946, contains a single-stranded RNA nuclease PIN domain [16], which is commonly found in toxins from bacterial toxin-antitoxin operons [22]. This gene also encodes a PhoH ATPase domain. This gene will be referred to as PIN-PhoH from here on. The second gene, SYNW1947, has a DNA binding domain which is a common feature of antitoxins [26]. The transcript levels of these genes remain unchanged for 1.5–3 h after Syn9 infection. All four of these genes are located in genomic islands that appear to be important in mediating the cyanobacterial response to phage infection [8,16].

We hypothesized that if these genes are defense related, their inactivation in the host would lead to a shortening of the infection cycle and/or an increase in phage progeny production. Here, we report that, indeed, the PIN-PhoH mutant produced more Syn9 progeny than the wild-type host. However, contrary to our expectations, the DUF3387 mutant produced a lower yield of phage progeny, suggesting that this two-gene operon is beneficial to the phage in the wild-type host.

## 2. Materials and Methods 

### 2.1. Growth of Cultures

The *Synechococcus* sp. WH8102 wild-type and mutant strains were grown in artificial seawater medium (ASW) [27], with modifications as described in Lindell et al. [28]. The cultures were grown at 22 °C under cool white light with a 14:10 h light:dark cycle at an intensity of 30 μmol photon·m^−2^·s^−1^ during the light period. These are the same culturing conditions as previously used [16], except that the culture volumes were 30 mL in this study (rather than 800 mL). Growth in liquid medium was monitored by measuring chlorophyll *a* autofluorescence as a proxy for biomass using a Turner Designs 10-AU flourometer (excitation/emission: 340–500/>665 nm) (Turner, San Jose, CA, USA) or the BioTek Synergy 2 microplate reader (excitation/emission: 440 ± 20/680 ± 20 nm) (BioTek, Winooski, VT, USA). 

Growth on semi-solid medium to produce colonies was done using a pour plating method [29,30,31]. Cells were mixed with medium containing Invitrogen Ultra Pure low melting point (LMP) agarose (ThermoFisher Scientific, Waltham, MA, USA) at a final concentration of 0.28%, poured into plastic petri dishes, and grown under the conditions described above. An antibiotic resistant heterotrophic helper strain, *Alteromonas* sp. strain EZ80, was added to the cells for plating colonies after conjugation (see below), to ensure a high plating efficiency [32]. 

The Syn9 phage lysate was prepared by infecting large volumes of *Synechococcus* WH8102. After complete lysis of the culture, cell debris was removed by centrifugation (13,131× *g* at 21 °C for 15 min) and filtration over a 0.2 μm filter (Nalge Nunc international, Rochester, NY, USA). The filtered lysate was concentrated 100-fold using Centricon Plus 70 centrifugal filters (100 kDa NMWL, Millipore, Billerica, MA, USA), to enable the infection of cultures with small (negligible) volume additions of the phage lysate.

### 2.2. Insertional Inactivation of Synechococcus WH8102 Genes

The predicted function of the conserved domains of the genes for inactivation was determined from conserved domain searches using the NCBI Blast conserved domains database (CDD) search and Pfam [16].

Insertional inactivation of the first gene in each of the two operons was done following Brahamsha [29]. An internal 192 bp fragment of the SYNW1659 gene was amplified by polymerase chain reaction (PCR) from *Synechococcus* WH8102 with primers that contain a *Bam*HI restriction site (shown in italics): SYNW1659ia2_FW (5′-*ATATATGGATCC*CTGCTGATCTGGCGGGTATTTG-3′) and SYNW1659ia2_Rv (5′-*ATATATGGATCC*GCCTTGGCAGACAACCCGTC-3′), and was cloned into the *Bam*HI site on the pMUT100 cargo plasmid. Due to the small size of this gene, the primers were designed to introduce stop codons on both sides of the SYNW1659 gene. For inactivation of the SYNW194*6* gene, an internal 350 bp fragment was PCR amplified from *Synechococcus* WH8102 using the following primers that also contain a *Bam*HI restriction site: SYNW1946ia_FW (5′-*ATATATGGATCC*CAGGCCCATGCTCTTGACGC-3′) and SYNW1946ia_Rv (5′-*ATATATGGATCC*AGCACCACGCCTTCATTTGC-3′), and was cloned into the pDS3 plasmid. The pMUT100 and pDS3 plasmids are derivatives of pBR322 that carry a kanamycin-resistance gene and can be mobilized into the *Synechococcus* WH8102, but cannot replicate in this host. pDS3 differs from pMUT100 in that the tetracycline gene was replaced with a chloramphenicol gene optimized for expression in *Prochloroccocus* [33]. The resulting plasmids were mobilized into *Synechococcus* WH8102 by conjugation using *Escherichia coli* MC1061, carrying the RP4 derivative conjugative plasmid pRK24 and the helper plasmid pRL528 as a donor [29]. Gene interruption occurs when the plasmid is integrated into the host chromosome by homologous recombination through a single crossover event. Exconjugants were selected for kanamycin resistance (25 µg·mL^−1^) on semi-solid medium. Verification of the complete segregation of chromosomes in the mutant (i.e., the absence of an intact gene in all of the chromosome copies) was done by PCR using primers which flank the target gene: SYNW1659ia_Fw (5′-ATATATGGATCCTCGCCCAAGGTCTCTGCCTG-3′) and SYNW1659ia_Rv (5′-ATATATGGATCCAGAGGAACTGGAGCGTGGCG-3′) for SYNW1659 and IAver_02_1946_Fw (5′-GATGCCTTGCCGATGGTGTTC-3′), and IAver_02_1946_Rv (5′-GTTTCCTTGACGCCGGGCAAG-3′) for SYNW1946. Verification that the plasmid was inserted at the desired location in the *Synechococcus* chromosome was done using one primer within the vector: pMUT_tet218F (5′-GCCCAGTCCTGCTCGCTTCG-3′), and one of the above verification primers within the chromosome external to the target gene [34].

### 2.3. Characterization of Infection Dynamics

One-step-growth curves of the Syn9 phage were carried out on exponentially growing cultures (30 mL) of each inactivation mutant, as well as the wild-type strain at the same cell concentration (~2 × 10^7^ cells·mL^−1^) without antibiotic selection. Syn9 was added to the cultures at a multiplicity of infection (MOI) of three infective phages per cell. For determination of the length of the latent period and lytic cycle, phage DNA in the extracellular medium was measured from samples collected every two hours from 0 to 12 h, as well as at 5 h after phage addition. For characterization of the replication of phage DNA inside the cell, intracellular phage DNA was measured from samples collected at 0, 0.5, 1, 2, 3, 4, 5, 6, and 8 h after phage addition. 

### 2.4. Quantification of Intracellular and Extracellular Phage Genomic DNA

Intracellular and extracellular phage genomic DNA (gDNA) was quantified using quantitative real-time PCR (qPCR), as described previously [8]. Extracellular phage gDNA was determined from filtrates containing phage particles after filtration over a 0.2 μm Acrodisc Syringe Filter (Pall Corporation) and dilution 100-fold in 10 mM Tris pH 8. Aliquots of 10 μL were frozen at −80 °C in triplicate and used directly for qPCR assays (see below). Intracellular phage DNA was determined from cells collected on 0.2 μm pore-sized polycarbonate filters (GE Healthcare Life Sciences, Boston, MA, USA) by filtration at a vacuum pressure of 7–10 inch Hg. Filters were washed three times with sterile seawater, once with 3 mL preservation solution (10 mM Tris, 100 mM EDTA, 0.5 M NaCl; pH 8), and were frozen at −80 °C. The DNA was extracted from the cells using a heat lysis method [35]. The polycarbonate filter with the cells was immersed in 10 mM Tris pH 8, and agitated in a mini-bead beater for 2 min at 5000 rpm, without beads. The sample was removed from the shards of filter, heated at 95 °C for 15 min, and 10 μL was used in triplicate qPCR reactions. 

### 2.5. Quantitative PCR Protocol

Assays for qPCR were carried out for the Syn9 portal protein gene (*g20*), as described previously [16]. Each qPCR reaction contained 1× Roche universal probe library (UPL) master mix (LightCycler^®^ 480 Probes Master, Roche, Penzberg, Germany), 100 nM UPL84 hydrolysis probe (Roche), 200 nM of HPLC-purified primers (Syn9_gp20_UPL84_F: 5′-TCGTTTAGAAACAGAAACCACATTT-3′ and Syn9_gp20_UPL84_R: 5′-AACTTTTGGAATTTAACTTCGTCAC-3′), and a 10 μL template in a total volume of 25 μL. Reactions were carried out on a LightCycler 480 Real-Time PCR System (Roche). The cycling program consisted of an initial denaturation step of 95 °C for 15 min followed by 45 cycles of amplification, each including 10 s of denaturation at 95 °C, a 30 s combined annealing and elongation step at 60 °C, and a fluorescence plate read (Ex/Em 465/510 nm). The crossing point was used to determine the amount of initial target using the absolute quantification/2nd-derivative maximum analysis with the LightCycler 480 software (release 1.5.0) (Roche). For intracellular gDNA determination, standard curves were produced using a serial dilution of purified phage DNA of a known quantity. For extracellular gDNA determination, standard curves of phage particles in 10 mM Tris pH 8, that had been enumerated by epifluorescence microscopy after SYBR staining [36], were used.

### 2.6. Burst Size and Virulence Determination

Burst size and virulence assays were carried out as described by Kirzner et al. [37]. Exponentially growing cultures were diluted to the same concentration (≈4 × 10^7^ cells per mL) and infected with the Syn9 phage at MOI = 3 in the morning hours. At 4 h after infection, when maximal adsorption had occurred (≈90%), but before the end of the latent period and the onset of cell lysis, the cultures were diluted 1000-fold and single cells were dispensed into individual wells of 96 well-plates using the FACSAria-IIIu cell sorter (Becton Dickenson, Franklin Lakes, NJ, USA). For the virulence assay, the cells were dispensed into wells containing the host culture (100 µL). The plates were incubated in growth conditions and chlorophyll *a* fluorescence was measured daily using the Synergy 2 microplate reader (BioTek). Lysis was determined by a significant decrease in fluorescence relative to the control plate containing only the host culture. The relative number of cleared wells in the infected versus the control plate is the percentage of cell lysis caused by the phage. For the burst size assay, cells were dispensed into individual wells containing only growth medium and incubated in growth conditions for 16–18 h after sorting. This allowed sufficient time for the completion of the infection cycle and the exit of all phage progeny. The contents of each well were then plated on a lawn of host cells and the number of plaques produced over a 10 day incubation period was monitored and indicated the number of progeny phages produced by that particular cell. Burst size was determined from plaque-containing plates (with more than one plaque) from four independent experiments for each strain. Each biological replicate consisted of phages arising from 60 to 96 cells each. 

### 2.7. Statistical Analysis

In order to test the significance of the differences between the results obtained for the wild-type and mutant strains (for growth rate, virulence, average burst sizes, and phage gDNA assays), a two tailed *t*-test for independent samples was carried out. This was done after ensuring that the data were normally distributed (*p* > 0.05) using the Kolmogorov-Smirnov or Shapiro-Wilk tests. A repeated measure ANOVA was used to assess whether significant differences existed in the timing of different stages of the infection cycle during Syn9 infection of the mutants relative to the wild-type strains. Since there were significant differences in the level of genomic DNA replication, that data were normalized to maximal levels in each strain before testing for differences in timing. The PASW statistics 17 package was used for these analyses (Rel. 17.0.3. September 2009. Chicago, IL, USA: SPSS Inc.).

## 3. Results

In order to investigate the effect of host response genes on the infection cycle, we generated two independent *Synechococcus* WH8102 mutants by the insertion of an antibiotic-carrying plasmid into the gene of interest by a single crossover [29] (see Methods). This physically interrupts the gene, rendering it inactive. For two-gene operons, such as in both of our cases, this insertion is expected to also prevent transcription of the downstream gene as it becomes separated from the promoter by the plasmid. Thus, the results presented in this study for each mutant likely relate to the effective inactivation of both genes in the two-gene operons. For simplicity, however, we refer to the mutants by the name of the insertionally inactivated gene: DUF3387 for SYNW1659-SYNW1658 and PIN-PhoH for SYNW1946-SYNW1947.

**Figure 1:**
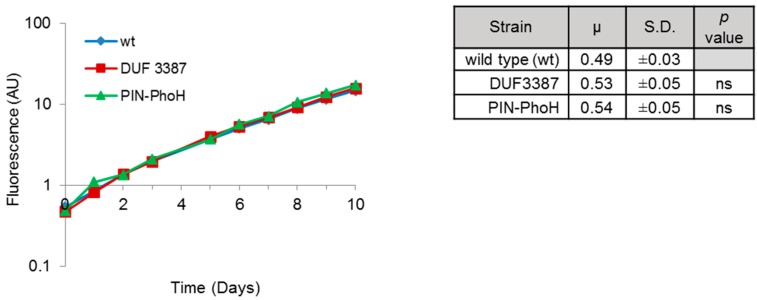
Growth of wild-type and mutant strains of *Synechococcus* WH8102. Representative growth curves are shown on the left and a table on the right presents the mean and standard deviation (S.D.) of the specific growth rate of four biological replicates. No significant differences were found in growth rates between the mutants (DUF3387 and PIN-PhoH) and the wild-type (wt) strains, nor between the two mutants.

Before investigating the effect of the insertional inactivation of the DUF3387 and PIN-PhoH genes on phage infection, we tested whether they affected the growth rate of the mutants under normal growth conditions. This was important since the efficiency of phage replication can be intimately linked to the growth rate of its host [8,38,39]. No significant differences were found between the growth rate of the mutants relative to the wild-type strain, nor were there differences in growth between the two mutants (Figure 1). Therefore, any differences observed in the Syn9 infection process in the two mutant strains relative to the wild-type strain cannot be attributed to intrinsic differences in host growth. 

**Figure 2:**
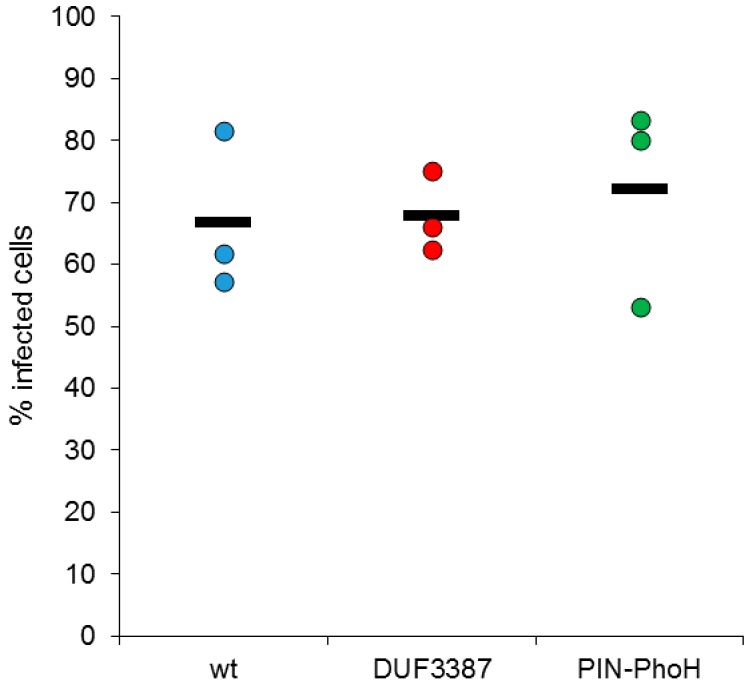
Virulence of the Syn9 cyanophage on wild-type and mutant strains of *Synechococcus* WH8102. The percentage of infected cells that were lysed in cultures of the two mutant strains (DUF3387 and PIN-PhoH) were compared to the wild-type (wt) strain. No significant differences were found. The bar denotes the mean of three biological replicates.

We began our investigation of the impact of the host mutations on the phage infection process by assessing phage virulence, as determined from the ability of the phage to infect and lyse the different host strains [37]. This was determined from the percentage of cells lysed by the Syn9 phage when infecting each of the inactivation mutants compared to infection of the wild-type *Synechococcus* strain. Virulence was not significantly different in either of the mutants relative to the wild-type strain (*n* = 3) and was approximately 70% for all three strains (Figure 2). This suggests that mutations in the DUF3387 and PIN-PhoH genes do not impact the ability of the phage to infect the *Synechococcus* host.

**Figure 3:**
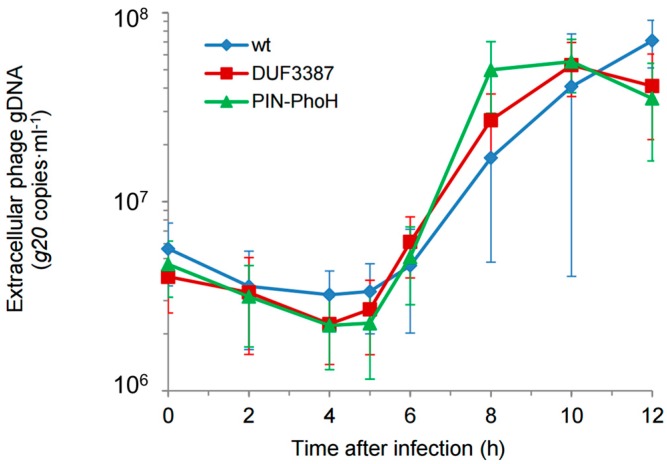
Infection dynamics of the Syn9 phage on wild-type and mutant strains of *Synechococcus* WH8102. One-step growth curves of the Syn9 phage were carried out to determine the length of the latent period and the lytic cycle during infection on the two mutant strains (DUF3387 and PIN-PhoH) and the wild-type (wt) strains. No differences were found in the timing of the infection cycle when comparing the Syn9 infection of the two mutant strains relative to its infection of the wild-type strain (*p* = 0.289 for DUF3387 and *p* = 0.071 for PIN-PhoH, each compared to the wt). Extracellular phage concentrations were determined from qPCR of the *g20* phage gene. Average and standard deviation of six biological replicates. gDNA: genomic DNA.

Next, we determined the effect of the host mutations on the length of the Syn9 lytic cycle. One-step-growth curves were carried out by determining the timing of phage release using a qPCR assay for the Syn9 portal protein gene (*g20*) in the extracellular medium. The length of the phage latent period was 5 h and the length of the lytic cycle was 8–10 h during infection of the two mutants, as well as during infection of the wild-type strain (Figure 3). These results are typical of previous findings for Syn9 on *Synechococcus* WH8102 [16]. Thus, the inactivation of the DUF3387 and PIN-PhoH genes did not affect the length of the phage infection cycle.

**Figure 4:**
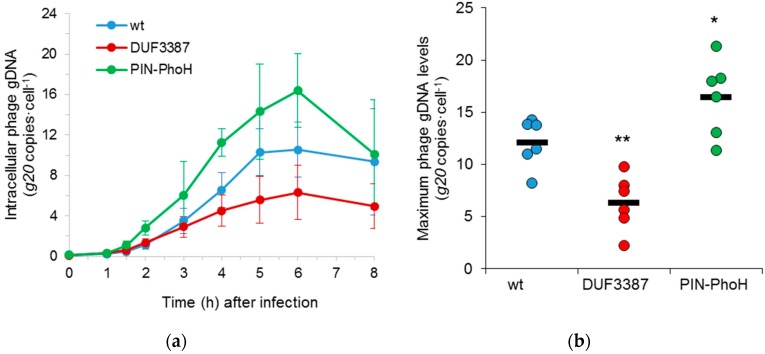
Intracellular phage gDNA replication during infection of wild-type and mutant strains of *Synechococcus* WH8102. (**a**) The timing and level of intracellular Syn9 genomic replication (determined by qPCR for the *g20* portal protein gene and normalized per cell) during infection of the two mutant (DUF3387 and PIN-PhoH) and wild-type (wt) strains. Mean and standard deviation of six biological replicates. No differences in the timing of DNA replication were found during the first 6 h of infection of the mutant strains relative to the wild-type strain (*p* = 0.61 for DUF3387 and *p* = 0.125 for PIN-PhoH, each compared to the wt). (**b**) Syn9 gDNA yield per host cell at the maximum amount of phage gDNA produced in that strain. The yield of Syn9 gDNA produced in the mutant strains was compared to the that for the wild-type strain. (* *p* < 0.05, ** *p* < 0.01). The bar denotes the mean of six biological replicates.

Following this, we asked whether the extent and timing of phage genome replication was altered during infection of the mutant strains. We analyzed phage genome replication, using the same qPCR assay as above, but on intracellular DNA extracted from infected cells. Here, the timing of phage DNA replication in the mutants was similar to that found in the wild-type host, beginning 1–2 h after phage addition (Figure 4a). However, clear differences in the number of phage genome copies were apparent for both mutants. Significantly more Syn9 phage genome copies were replicated in the PIN-PhoH mutant than in the wild-type strain (*p* < 0.05, *n* = 6) (Figure 4b). In contrast, the phage gDNA levels were significantly lower in the DUF3387 mutant relative to the wild-type strain (*p* < 0.01, *n* = 6) (Figure 4b).

**Figure 5:**
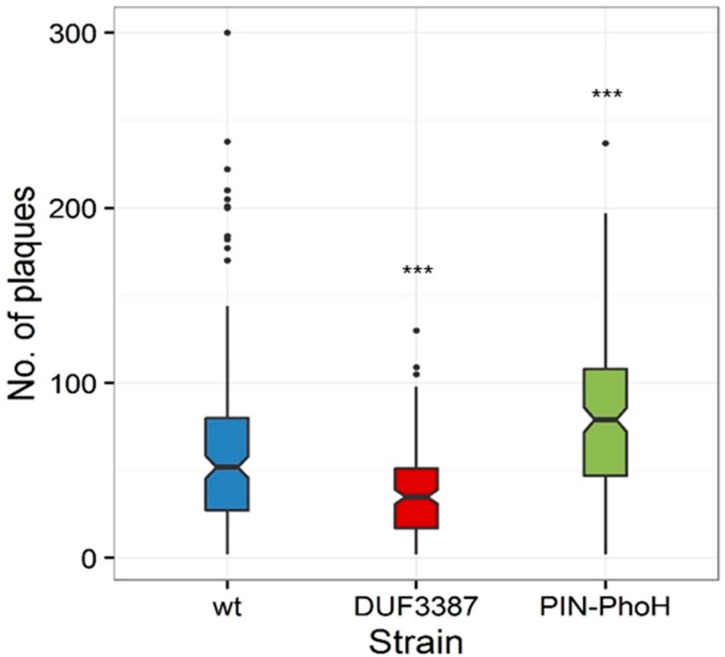
Distribution of the number of infective Syn9 phages produced per cell when infecting the wild-type and mutant strains of *Synechococcus* WH8102. Box plot of single cell burst sizes. Burst sizes were significantly lower on the DUF3387 mutant than on the wild-type (wt) strain (*p* < 0.001, *n* = 164 cells for DUF3387 mutant and 174 cells for the wild-type strain), but were significantly higher on the PIN-PhoH mutant than on the wild-type strain (*p* = 0.001, *n* = 189 cells for the PIN-PhoH mutant and 174 cells for the wild-type strain). The middle line of the box plot denotes the median burst size and the boxes surrounding the median correspond to the 25th (lower) and 75th (upper) percentiles. Outliers are plotted as individual points. *** *p* ≤ 0.001.

In order to assess whether these differences in phage genome replication translated into changes in phage fitness, we investigated the number of infective phages produced per cell using a single-cell burst size assay [37]. Similar to phage genome replication, the median burst size of the Syn9 phage on the PIN-PhoH mutant (79 phages·cell^−1^) was significantly higher than on the wild-type host (52 phages·cell^−1^) (*p* = 0.001, *n* = 189 cells for the PIN-PhoH mutant and 174 cells for the wild-type host) (Figure 5). In contrast, the median burst size of Syn9 on the DUF3387 mutant (35 phages·cell^−1^) was significantly lower than that found for the wild type strain (Figure 5) (*p* < 0.001, *n* = 164 cells of the DUF3387 mutant). These findings suggest that the product of the PIN-PhoH gene serves the wild-type host during infection by restraining phage genome replication and phage progeny production. However, the lower phage gDNA levels and smaller burst size in the DUF3387 mutant suggests that, in this case, the host gene assists phage genome replication and progeny production when infecting the wild-type host.

Our findings indicate that the insertional inactivation of the two genes that responded transcriptionally to Syn9 infection in the wild-type strain [16] impacts phage fitness. This was manifested at the level of phage genome replication and the number of infective phage progeny produced per cell. However, these mutations had no effect on the ability of the phage to infect the host, nor did they impact the timing of the infection process.

## 4. Discussion

Over the past decade, a number of whole-genome transcriptional studies have shown that phage infection causes a discernable transcriptional response in different bacterial hosts [17,18,19,20,40,41], including in marine cyanobacteria [8,16]. Previously, though, it was thought that phage infection led to a complete and immediate shut-down of host transcription [42,43]. It has been suggested, from homology-based annotations, that some of these genes function as host defenses against infection, while others may be utilized by the phage to enhance reproduction [8,16,20,40,41]. However, the function of such host response genes during infection and their impact on the infection process has rarely been tested (but see [44,45]). Here, we show that at least two cyanobacterial response genes influence the phage infection process at the stage of phage genome replication and impact phage fitness. 

The induction of such host-response genes could be the cyanobacterial cell’s attempt at defense against phage infection. We initially hypothesized that this is the case for the two sets of genes investigated in this study since they have, or are associated with, domains found in known bacterial defense systems against phage infection. The potential toxin-antitoxin system in *Synechococcus* WH8102, as exemplified by the PIN-PhoH operon (SYNW1946 and SYNW1947), is an example in hand. Our findings for the PIN-PhoH mutant support this hypothesis as it produced significantly more phages than the wild-type strain and thus, this operon likely limits phage replication in the wild-type *Synechococcus* WH8102. Similar findings were reported for the P1 phage in a deletion mutant of the *mazEF* toxin-antitoxin system in *E. coli* [25] and the expression of the *hok/sok* system on a plasmid in *E. coli* led to a reduced burst size of T4 [24]. 

The vast majority of PIN-domain containing proteins are the toxic components of toxin-antitoxin operons in bacteria [22]. These PIN domains function as sequence specific single stranded RNases [46]. Recently, a PIN-PhoH protein was found to be the RNase toxin in a toxin-antitoxin system from *Myocbacterium tuberculosis* [23]. Thus, the PIN-PhoH protein in *Synechococcus* WH8102 may limit phage progeny production by acting as an RNase toxin which uses the energy provided from the hydrolysis of ATP by the PhoH ATPase domain to cleave phage RNA during infection. Since the mutant was found to impact phage genome replication, the RNA target of the protein may be mRNA needed to produce replication proteins or perhaps the RNA primers required for replication itself. It should be noted that these PIN-PhoH genes are distinct from the *phoH* genes found in many bacterial and phage genomes, including those of cyanobacteria and cyanophages. 

If indeed the PIN-PhoH protein is a defense system in *Synechococcus* WH8102, it is ultimately unsuccessful against Syn9 as this phage kills the wild-type host, and no increase in the number of cells killed (its virulence) was observed in the mutant. It is possible that the Syn9 phage encodes genes that interfere with the activity of this toxin-antitoxin system, as is known for T4 [47,48], although no evidence currently exists to support this possibility in Syn9. Furthermore, it may be more successful in defense against other phages. A homology search found that homologues of the same two genes arranged in the same order are also found in *Synechococcus* WH8109 and CC9605, but these genes were not part of the host response gene repertoire during *Synechococcus* WH8109 infection by the Syn9 phage [16].

Unlike the PIN-PhoH operon, our results argue against the DUF3387 operon (SYNW1659 and SYNW1658 operon) being a host defense mechanism. The lower yield of phage progeny produced on the DUF3387 mutant than on the wild-type suggests that these genes facilitate and increase the phage yield in the wild-type *Synechococcus*. While we do not know the mechanism by which this gene enhances phage reproduction, the association of the DUF3387 domain with helicase genes (also beyond those found in restriction enzymes), coupled with the decrease in phage genome levels in the mutant, provides the intriguing possibility that this gene is directly involved in the process of DNA replication. Thus, this gene may be induced as part of the cyanobacterium’s stress response and is exploited by the phage for its replication or is directly upregulated by the phage. Our findings do not allow us to discern between these two possibilities. However, it is well known that cellular stress response proteins are utilized by various phages for their replication in *E. coli* [49,50,51,52] . 

These findings were initially surprising as the DUF3387 domain is associated with type I and type III restriction enzymes, well established as potent mechanisms of defense against phage infection that degrade unmodified phage DNA upon entry into the bacterium (see review in Labrie et al. [21]). However, other domains carry out the endonucleotyic activity of restriction enzymes [53]. Thus, this domain, whose function remains unknown, appears to aid the phage when it is disconnected from the endonucleolytic domain. 

Alternatively, the phenotype of this mutant may not be related to the restriction enzyme associated domain, but to the adjacent gene, SYNW1658, which encodes a different domain of unknown function (DUF1651). Intriguingly, this protein domain is limited to, but widespread, in marine cyanobacteria among the bacteria, but is also found in a single known coliphage (the ECBP5 podovirus, NC_027330 [54]). Furthermore, five other DUF1651 domain containing genes in three different marine cyanobacteria are part of the host response to phage infection. These include two other *Synechococcus* WH8102 genes (SYNW2106 and SYNW1944) and a *Synechococcus* WH8109 gene (Syncc8109_0491) in response to Syn9 infection [16], as well as two genes in *Prochlorococcus* MED4 (PMM0684 and PMM0819), whose transcript levels increase in response to infection by the T7-like podovirus, P-SSP7 [8]. It thus appears likely that DUF1651 domain containing proteins are important for cyanophage during infection, and may be responsible for increasing the yield of infective cyanophage in multiple distinct marine cyanobacterial hosts. Why such a gene would be retained by cyanobacteria is unclear. Perhaps it is a stress response gene that provides an advantage to the cell when exposed to other stressors that the phage has evolved to utilize.

## 5. Conclusions

The findings presented here indicate that host-response genes play a functional role in the phage infection process. They further show that this functionality is not unidirectional: In certain cases, they are in service of the host as an attempt at defense against infection. In other cases, however, they can be exploited by the phage, even though their role in the host may well be a bona-fide response to the abiotic or biotic stressors that they are exposed to in the oceans. Since these genes are located in genomic islands and are often part of the flexible genome, these results continue to highlight the importance of such genomic regions and their gene content for host-phage interactions and the coevolutionary process between cyanobacteria and the phages that infect them.
